# Immune responses during neoadjuvant chemotherapy in triple negative breast cancer

**DOI:** 10.17179/excli2020-2869

**Published:** 2020-09-11

**Authors:** Ahmed Ghallab

**Affiliations:** 1 Forensic Medicine and Toxicology Department, Faculty of Veterinary Medicine, South Valley University, Qena, Egypt

## ⁯⁯

***Dear Editor,***

Recently, Axelrod and colleagues analyzed immune-related gene expression changes in breast cancer before and after neoadjuvant chemotherapy (Axelrod et al., 2020[1]). The authors analyzed 83 breast cancer tissue specimens, among them 44 triple negative carcinomas. An interesting result was that upregulation of immune-related genes was associated with better prognosis in the triple negative breast carcinomas (Axelrod et al., 2020[1]). Moreover, the authors studied PD-1^Hi^ CD8+ peripheral T-cells for a cytolytic gene signature. Expression of cytotoxic genes in the peripheral blood was associated with worse prognosis, which may be explained by persistent micrometastatic disease that finally leads to recurrence (Axelrod et al., 2020[1]). 

Numerous studies have analyzed the association of breast cancer with outcome, including the spontaneous course of disease and response to chemotherapy (Sparano et al., 2018[16]; Wang et al., 2018[19]; Finak et al., 2008[6]; van de Vijver et al., 2002[18]; Kwa et al., 2017[9]). High levels of T-cell and also B-cell/plasma cell associated genes were observed in breast carcinomas with relatively good prognosis (Schmidt et al., 2008[12], 2012[13], 2018[14]; Heimes et al., 2017[7]) but also in other tumor types (Edlund et al., 2019[5]; Lohr et al., 2013[10]). In contrast, antioxidative genes (Cadenas et al., 2010[2]) and genes associated with proliferation (Siggelkow et al., 2012[15]; Hellwig et al., 2016[8]; Cadenas et al., 2014[3]) and lipid (Cadenas et al., 2019[4]) as well as glycerophospholipid (Marchan et al., 2017[11]; Stewart et al., 2012[17]) metabolism are linked to worse prognosis. The current study of Axelrod et al. (2020[1]) is of high interest, because the authors systematically analyzed tumor tissue before and after chemotherapy and clearly demonstrated the prognostic role of genes associated with better response. 

## Conflict of interest

The author declares no conflict of interest.
